# The association between risk of limb fracture and type 2 diabetes mellitus

**DOI:** 10.18632/oncotarget.23937

**Published:** 2018-01-05

**Authors:** Jie Liu, Lei Cao, Yao-Wen Qian, Zhi-Xin Chen, Shi-Fang Guo, Wei-Qiang Sun, Zong-Ru He

**Affiliations:** ^1^ Orthopaedic Department of People's Hospital of Gansu Provincial, Lanzhou 730000, China; ^2^ Pediatrical Department of Gansu Provincial Maternity and Child-care Hospital, Lanzhou 730000, China; ^3^ Traditional Chinese Hospital of Gansu Zhang County, Dingxi 743000, China

**Keywords:** limb fracture, diabetes mellitus, risk factor, meta-analysis

## Abstract

**Background:**

Recently, increasing reports showed that the risk of fracture may be correlated with type 2 diabetes mellitus (T2DM). However, their results still remained controversial. Thus we performed a meta-analysis including 11 studies to estimate the risk factor of limb fracture in type 2 diabetes mellitus.

**Materials and Methods:**

Databases including PubMed, Embase, Cochrane Library and Web of Science were searched to September, 2017. Risk Ratio (RR) with its 95% confidence intervals (CI) was used to evaluate the association between risk of limb fracture and type 2 diabetes mellitus. Two reviewers assessed the quality of all the included studies and extracted data for analysis independently.

**Results:**

A total of 11 studies including 663,923 participants were included in this meta-analysis. Our analysis results showed that patients with type 2 diabetes mellitus had a significant association with risk of limb fracture (RR 1.18; 95% CI 1.02–1.35), including leg or ankle fracture (RR 1.80; 95% CI 1.13–2.87). Subgroup analysis showed individuals with type 2 diabetes had almost two-fold excessive risk of leg/ankle fracture in women and the pooled RR of leg/ankle fracture was 2.03 (95% CI 1.36–3.05; *P* = 0.0006).

**Conclusions:**

The results of the present meta-analysis showed that individuals with type 2 diabetes mellitus had higher risk of limb fractures, and this relationship is more pronounced in leg or ankle fracture.

## INTRODUCTION

Fractures account for a large proportion in the global disease burden. At present, one of the most common risks of fracture should be osteoporosis, which lead a significant mortality, morbidity and socioeconomic burden in elderly patients [1]. However, recently, increasing studies have reported that diabetes mellitus was associated with an increased risk of fracture and it has been observed that individuals with diabetes mellitus experienced significant higher incidence of fracture in both vertebral and non-vertebral or limb fracture [2–9]. The relative risk estimates may depend on the age and gender distribution of the population in question [10]. Bone mineral density and the fracture risk assessment tool at present seems not explain the increased fracture incidence in patients with diabetes [11–14]. Shared risk factors as pancreatitis, alcohol use, smoking and oral glucocorticoids may influence the observed fracture risk in patients with diabetes. Fractures usually compel individuals experiencing long-term disability and serious socioeconomic burden, even increased mortality risk and poor functional recovery [1, 15]. Therefore, it is crucial to identify the modifiable risk factors for individuals and prevent or decrease it early.

At present, many studies have showed that diabetes mellitus patients usually experienced more opportunity of hip, pelvis, proximal humerus, distal forearm, ankle, knee, foot, wrist and vertebral fractures [16–25]. However, fallaciously, their results still remained inconsistent, with several studies drawing inverse conclusions. Thus, the present meta-analysis was designed based on relevant studies to analyze and evaluate the association between risk of limb fracture and type 2 diabetes mellitus. The purpose of the present analysis we performed was to explore the risk factor of limb fracture in type 2 diabetes mellitus and to clear if diabetes is one of independent risk factors of limb fractures. Further, we also try to analysis the risk of limb fractures in different fracture site and gender.

## RESULTS

### Included studies, study characteristics and study quality

**Figure 1:**
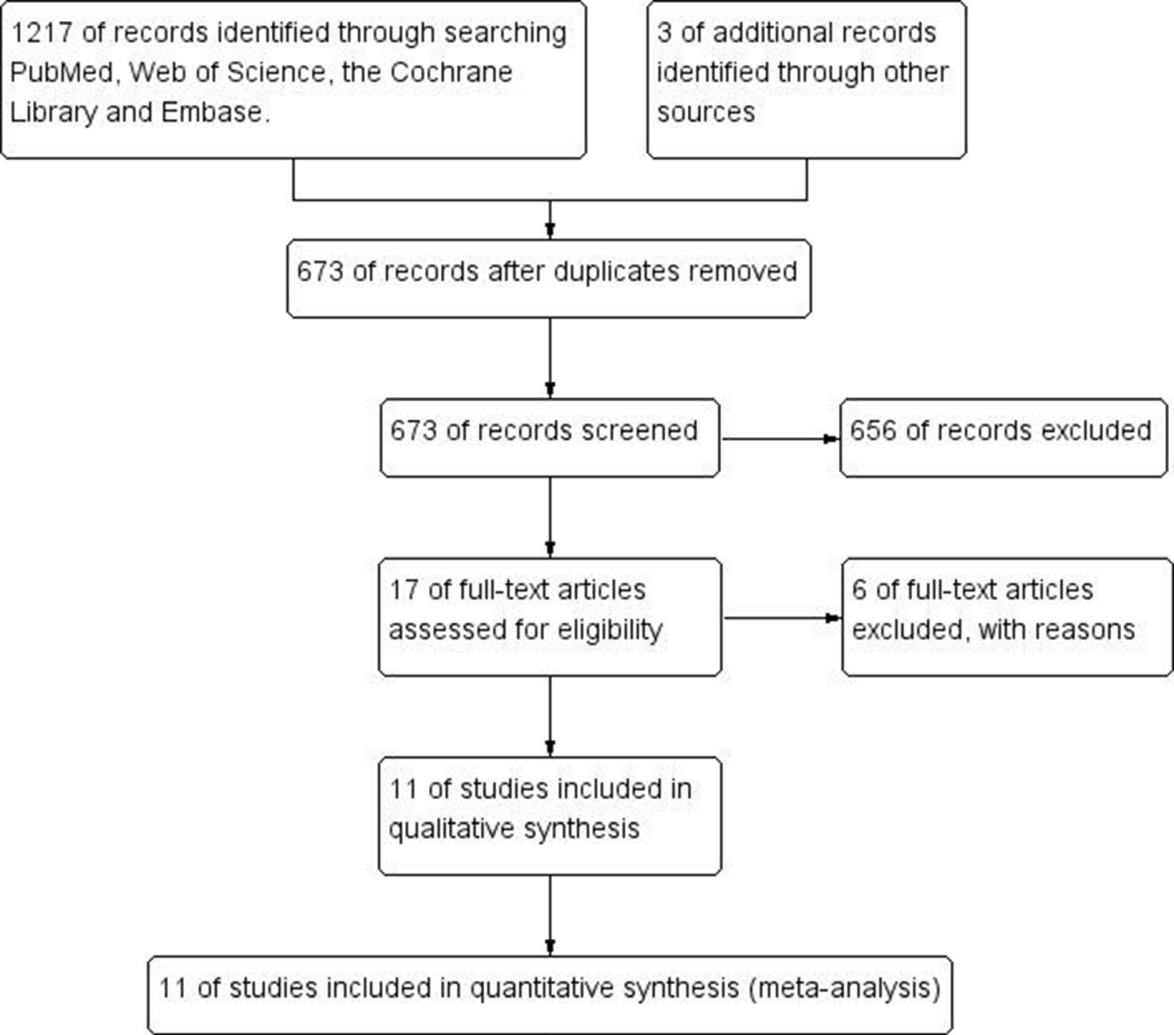
Flow diagram following the PRISMA template of the search strategy for studies included in this meta-analysis

**Figure 2:**
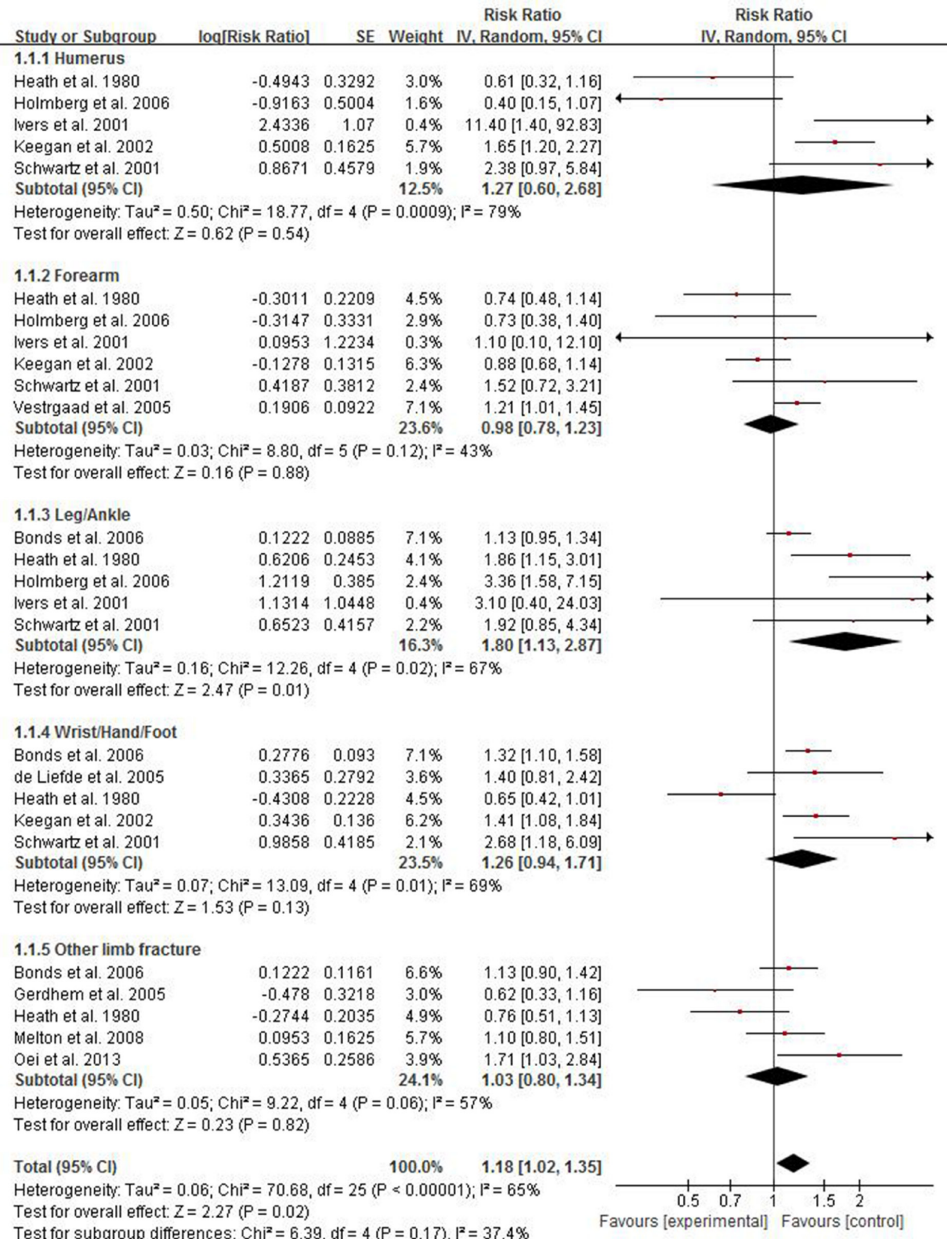
Forest plot showing the pooled results of groups or subgroups for the association between risk of limb fractures and type 2 diabetes mellitus in both of women and men

673 studies were retrieved after duplicated records removed from the preliminary screening of 1220 titles. By screening the titles and abstracts of the retrieved records, 17 studies of full texts were considered for further evaluation after 656 of records excluded. Six full articles were excluded at the final stage of filtrating work for reasons: two for review studies [16, 22], one for letters [19], two for lack of available data [6, 14], one for not about limb fracture [2]. Eventually, total of 11 studies including 663,923 participants were included in this meta-analysis. In these included studies, two were case–control studies and nine were cohort studies. The sites of fracture in these studies included hip, pelvis, proximal humerus, distal forearm, ankle, knee, foot, wrist and vertebral. As shown in Figure 2, in the present meta-analysis, the results of five studies were combined in the subgroups for pooled analysis of proximal humerus, six for forearm, five for leg/ankle, five for wrist/hand/foot and five for other limb fractures. According to one of the most widely used criteria for the assessment of the quality of nonrandomized studies, NOS criteria, the majority of studies had high-quality. In these studies, five with NOS score of 7, three with NOS score of 6 and three studies with NOS score of 8 respectively. The detail search process and summary of studies features were shown in study flow diagram (Figure 1) and Supplementary Table 1.

### Association between risk of limb fractures and type 2 diabetes mellitus

There were eight studies providing data for estimating the association between risk of limb fractures and type 2 diabetes mellitus. As displayed in Figure 2, the pooled results showed that, compared with individuals without diabetes, individuals with type 2 diabetes mellitus had a higher risk of limb fracture, with the pooled RR being 1.18 (95% CI 1.02–1.35; *P* = 0.02). The analysis was estimated using a random-effect model for the significant heterogeneity (*p* < 0.00001, *I*^2^ = 65%).

Subgroup analysis was conducted to find which site of fracture had significant association with type 2 diabetes mellitus. As shown in Figure 2, individuals with type 2 diabetes had an excessive risk of leg/ankle fracture and the pooled RR of leg/ankle fracture was 1.80 (95% CI 1.13–2.87; *P* = 0.01). No significant results were observed in subgroups of humerus (RR 1.27; 95% CI 0.60–2.68), forearm (RR 0.98; 95% CI 0.78–1.23), wrist/hand/foot (RR 1.26; 95% CI 0.94–1.71) and other limb fractures (RR 1.03; 95% CI 0.80–1.34). Considering the significant intergroup heterogeneity, a random effect model was used.

### Association between risk of limb fractures and type 2 diabetes mellitus in women

**Figure 3:**
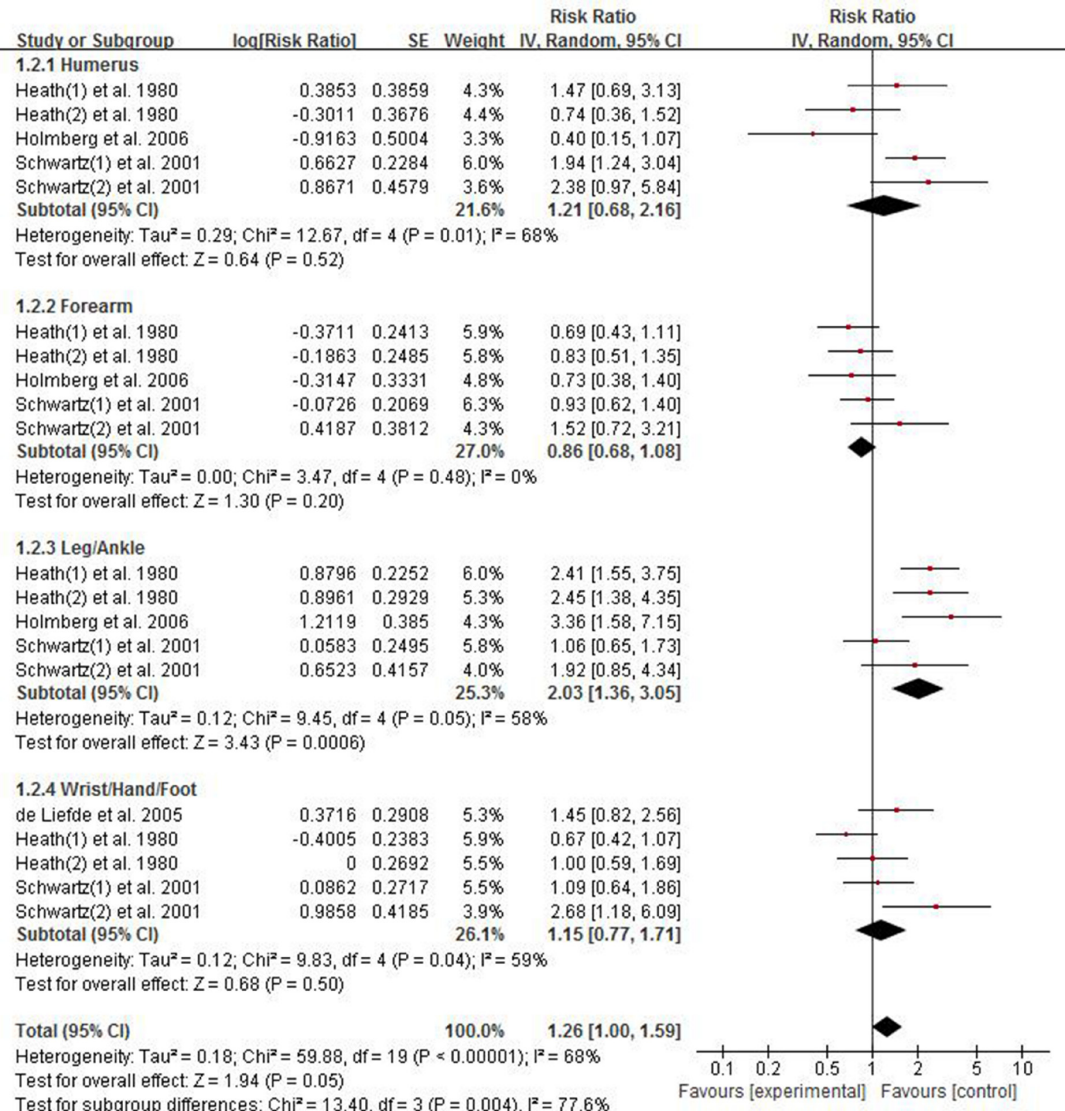
Forest plot showing the pooled results of groups or subgroups for the association between risk of limb fractures and type 2 diabetes mellitus in women

There were four studies providing data for estimating the association between risk of limb fractures and type 2 diabetes mellitus in women. As displayed in Figure 3, the pooled results showed that female individuals with type 2 diabetes mellitus had an excessive risk of limb fracture compared with one without diabetes, with the pooled RR being 1.26 (95% CI 1.00–1.59). Considering the significant heterogeneity (*p* < 0.00001, *I*^2^ = 68%), a random effect model was used to combine the results.

We also conducted subgroup analysis to find which site of fracture had significant association with type 2 diabetes mellitus in women. The results of subgroup analysis showed individuals with type 2 diabetes had almost two-fold excessive risk of leg/ankle fracture in women and the pooled RR of leg/ankle fracture was 2.03 (95% CI 1.36–3.05; *P* = 0.0006). No significant results were observed in subgroups of humerus (RR 1.21; 95% CI 0.68–2.16), forearm (RR 0.86; 95% CI 0.68–1.08), wrist/hand/foot (RR 1.15; 95% CI 0.77–1.71).

### Association between risk of limb fractures and type 2 diabetes mellitus in men

**Figure 4:**
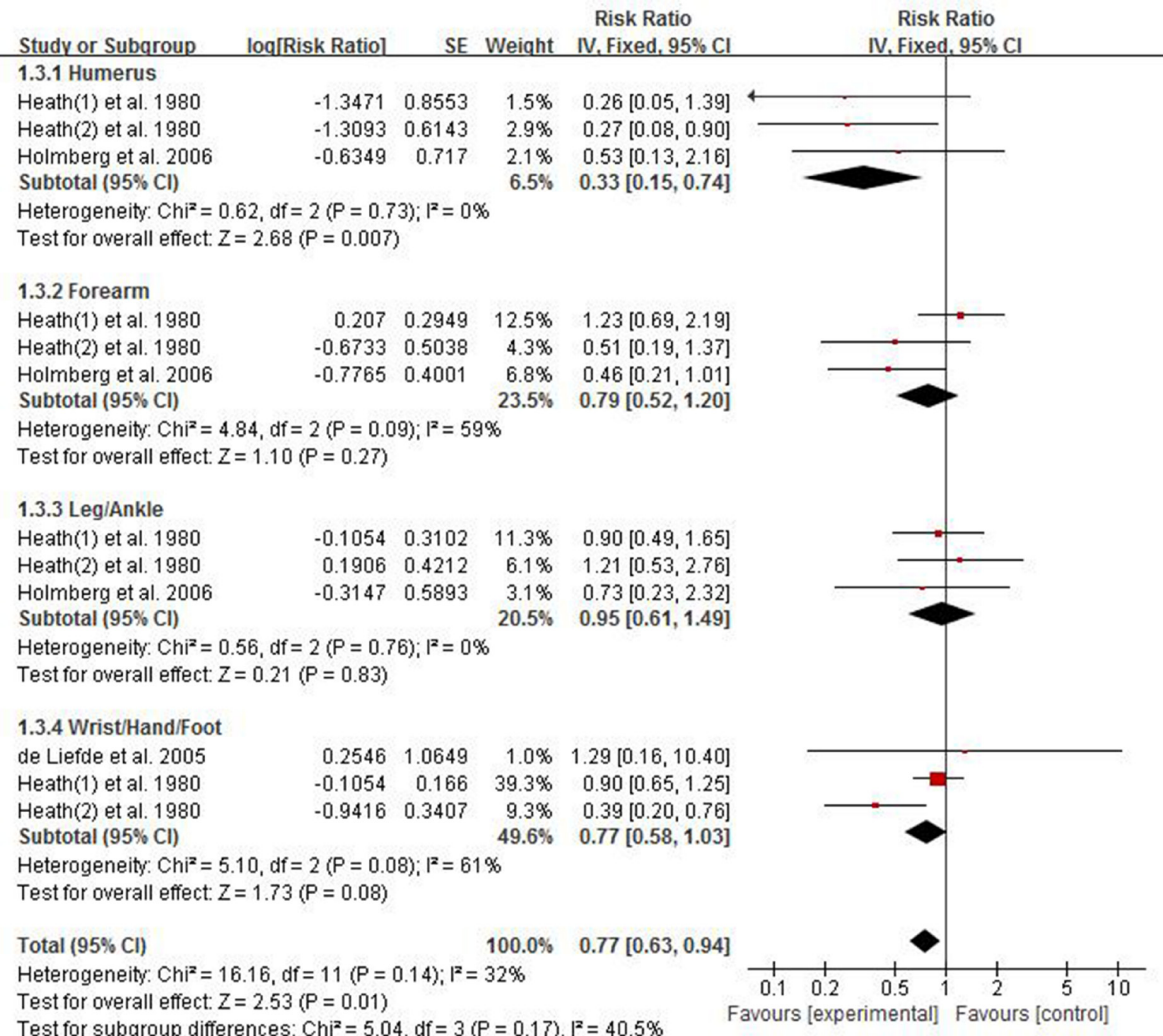
Forest plot showing the pooled results of groups or subgroups for the association between risk of limb fractures and type 2 diabetes mellitus in men

There were three studies providing data for estimating the association between risk of limb fractures and type 2 diabetes mellitus in men. As displayed in Figure 4, the pooled results inversely showed that male individuals with type 2 diabetes mellitus had a less risk of limb fracture compared with one without diabetes, with the pooled RR being 0.77 (95% CI 0.63–0.94). Considering no presence of heterogeneity (*p* = 0.14, *I*^2^ = 32%), a fixed-effect model was used to combine the results. The results of subgroup analysis showed individuals with type 2 diabetes had an less risk of humerus fracture in women and the pooled RR of humerus fracture was 0.33 (95% CI 0.15–0.74). No significant results were observed in subgroups of leg/ankle fracture (RR 0.95; 95% CI 0.61–1.49), forearm (RR 0.79; 95% CI 0.52–1.20), wrist/hand/foot (RR 0.77; 95% CI 0.58–1.03).

### Sensitivity analysis and publication bias

**Figure 5:**
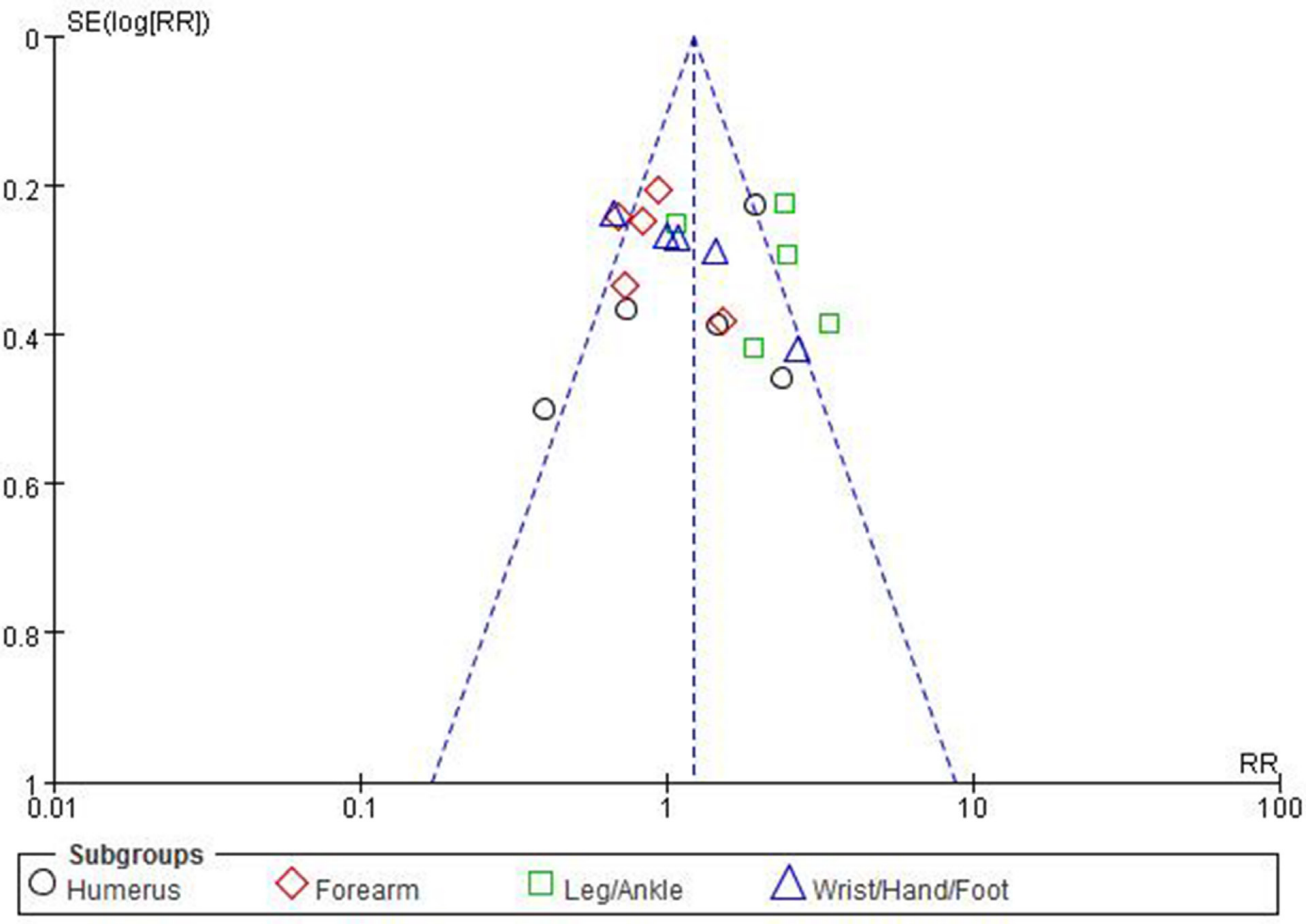
Funnel plots for detecting publication bias of the association between risk of limb fractures and type 2 diabetes mellitus

In order to examine the stability of the combined results, we performed sensitivity analysis. We conducted sensitivity analysis by omitting any single study then combining the rest of studies in the list. Sensitivity analysis showed that the combined RR of the association between risk of leg/ankle fracture and overall type 2 diabetes mellitus had high stability by omitting any single study. High stability was also found in the pooled result of risk of leg/ankle fracture in overall T2DM individuals. Sensitivity analysis indicated that there was none of any single study affecting the significance of combined RR of risk of leg/ankle fracture in T2DM individuals. However, for the association between risk of limb fractures and type 2 diabetes mellitus in men, sensitivity analysis showed that the study of Heath(2) et al. 1980 significantly affected the results of both overall group and subgroup, for that the pooled RR of overall group ranged from 0.77 (95% CI 0.63–0.94) to 0.86 (95% CI 0.68–1.08) and the pooled RR of subgroup of humerus ranged from 0.33 (95% CI 0.15–0.74) to 0.40 (95% CI 0.13–1.16) after omitting the study of Heath(2) et al. 1980. Funnel plots were conducted for assessing the publication bias of included literatures and we could roughly assess the publication bias by seeing whether their shapes were of any obvious asymmetry. The funnel plots showed no clear evidence of publication bias Figure 5.

## DISCUSSION AND CONCLUSIONS

In recent years, diabetes increasing the risk of fracture was increasingly given great interest. Up to the present, a great many studies have showed that diabetes mellitus patients usually experienced more opportunity of fracture in hip, pelvis, proximal humerus, distal forearm, ankle, knee, foot, wrist and vertebral. However, the mechanism between risk of fracture and diabetes has not been well studied to date. What’ more, it seems that the affection of diabetes is inequable in different site of fracture in different gender. For example, in study of Holmberg AH et al. [26], diabetes could affect the risk of low-energy fracture (RR 1.59; 95% CI 1.33–2.86), vertebral fracture (RR 3.56; 95% CI 1.75–7.23), ankle fracture (RR 3.36; 95% CI 1.58–7.15) and hip fracture (RR 4.07; 95% CI 1.79–9.26), instead of forearm fracture (RR 0.73; 95% CI 0.38–1.41) and proximal humerus fracture (RR 0.40; 95% CI 0.15–1.09) in women. However, for the risk of fracture in men, significant diabetic affecting was observed only in low-energy fracture (RR 2.50; 95% CI 1.75–3.57) and hip fracture (RR 7.75; 95% CI 4.37–13.7), but no in vertebral fracture (RR 0.85; 95% CI 0.27–2.65), ankle fracture (RR 0.73; 95% CI 0.23–2.29), forearm fracture (RR 0.46; 95% CI 0.21–1.04) and proximal humerus fracture (RR 0.53; 95% CI 0.13–2.13). Several meta-analyses have demonstrated that diabetes had significant association with risk of hip fracture, low-energy fracture and vertebral fracture [16–18, 20, 25], but there were controversy and inconformity in forearm fracture, proximal humerus fracture and other limb fractures.

Thus, the present meta-analysis we performed for purpose of demonstrating the association between type 2 diabetes mellitus and risk of limb fractures. Eleven studies including 663,923 individuals were included in this meta-analysis and we found two case–control studies and nine cohort studies. These studies involved hip fracture, pelvis fracture, proximal humerus fracture, distal forearm fracture, ankle fracture, knee fracture, foot fracture, wrist and vertebral fracture. To well study the association between limb fracture and T2DM, we only extracted and took advantage of data and information about humerus, forearm, ankle, knee, foot, hand, leg and wrist fracture. In addition, in order to clear which site of limb fracture had significant association with type 2 diabetes mellitus, we performed subgroup analysis. Our analysis results strongly supported the conclusions that individuals with type 2 diabetes mellitus had a higher risk of limb fracture, with the pooled RR being 1.18 (95% CI 1.02–1.35; *P* = 0.02). Subgroups analysis further showed that individuals with type 2 diabetes had an excessive risk of leg/ankle fracture and the pooled RR of leg/ankle fracture was 1.80 (95% CI 1.13–2.87; *P* = 0.01). Similar conclusion was also found in women. However, opposite results were found in male risk of limb fracture. To find the cause of this phenomenon, sensitivity analysis was conducted. Sensitivity analysis found that one study, Heath(2) et al. [9], significantly affected the results of both overall group and subgroup in men, for that the pooled RR of overall group ranged from 0.77 (95% CI 0.63–0.94) to 0.86 (95% CI 0.68–1.08) and the pooled RR of subgroup of humerus in men ranged from 0.33 (95% CI 0.15–0.74) to 0.40 (95% CI 0.13–1.16) after omitting the study of Heath(2) et al. [9]. In contrast, sensitivity analysis showed high stability in overall and women risk of fracture. In addition, compared with overall and women, the number of studies for men was lees, which may result in significant bias and unauthentic results. In the study of Holmberg AH, et al. [26], it was found that female diabetic individuals had significant three-fold excessive risk of ankle fracture, but not significant in men. Thus, future researches need to be designed to demonstrate this controversy further.

Several limitations for the present meta-analysis exist. The main limitation was due to the paucity of reported data. This meta-analysis included two case–control studies and nine cohort studies and both of them were retrospective observational researches. The majority of the studies we included were prospectively designed to measure the risk of fractures except the study of Schwartz AV, et al. 2001 [7]. Additionally, risk factors as age, pancreatitis, alcohol use, smoking and oral glucocorticoids may influence the observed fracture risk in patients with diabetes. In the study of Schwartz AV, et al. [7], the authors compared the risk of fracture among older women with diabetes, stratified by insulin use, compared with nondiabetic women. In the group of women with diabetes not using insulin compared with nondiabetics, significant result was found that women with diabetes not using insulin had a nearly two-fold excessive risk of proximal humerus fracture (RR 1.94; 95% CI 1.24–3.02). However, no significant result was found in risk of proximal humerus fracture in group of women with diabetes using insulin compared with nondiabetics. Therefore, it seems that using insulin to control blood glucose for diabetes may reduce the risk of proximal humerus fracture. The duration of diabetes may also be one of factors affecting the risk of fracture. Ivers RQ, et al [8]. reported that, compared with individuals with no diabetes, individuals with 0–4 and 5–9 years duration of diabetes had no significant rise of fracture risk, but there was obvious significance for individuals with more than 10 years duration of diabetes (RR 2.9; 95% CI 1.2–7.0). To our dismay, we had no sufficient data to determine whether the risk of fracture varied with the particular factors above. Thus, further studies should be designed to adjust all the possible covariates of fracture risk.

In conclusion, the results of the present meta-analysis showed that individuals with type 2 diabetes mellitus had excessive risk of limb fractures, and this association was more significant in leg or ankle fracture, especially in female diabetes mellitus.

## MATERIALS AND METHODS

### Including and excluding criteria

The including criteria of this meta-analysis were as follows: (1) Both cohort and case-control studies were included; (2) Studies about the association between T2DM and fracture risk (forearm, humerus, ankle, foot, wrist, hand, leg or other limb fractures); (3) RR with its 95% CI of association between T2DM and fracture risk were reported or can be obtained from studies.

Excluding criteria were as follows: (1) Trials on animals; (2) Abstracts, letters, editorials, expert opinions, reviews, conference records, case reports; (3) Participants included in studies having other disease which could result in fracture or could increase risk of fracture; (4) Studies without sufficient data; (5) Duplicate articles were excluded.

### Search strategy

We searched PubMed, Embase, Cochrane Library and Web of Science to September, 2017. We also searched the citation lists of included studies. Our searching terms and procedures were as follows: “fracture” AND “diabetes” AND “risk factors”. We searched the databases with these terms in English, including references of some literatures we read. Two assessors independently screened the titles and abstracts of each study. Once relevant studies became certain, the full texts were obtained for further evaluation.

### Quality assessment

Two reviewers assessed the quality of all the included studies using the 9-star Newcastle-Ottawa Scale (NOS) independently, and the total scores of each study were displayed in the characteristics table. The scores were judged according to the three aspects of NOS of evaluation: selection, comparability, and outcome between the case group and control group [27].

### Data extraction

Data for the analysis were extracted independently by two reviewers, and disagreement was resolved by their discussion. In addition, the extracted contents included study demographics, published years, country, trial design, cancer location, outcomes, using a standardized form. This study was performed strictly abiding by the standards of the Preferred Reporting Items for Systematic Reviews and Meta-Analyses (PRISMA) [28].

Data collected were input into Review Manager 5.2 software for analysis [29].

### Statistical analysis

In the present meta-analysis, the RR was used to evaluate the association between T2DM and risk of limb fracture. The associated 95% confidence intervals (CI) were also measured.

The heterogeneity between studies was evaluated by the chi-square-based *Q* statistical test, with *P* value and *I*^2^ statistic, ranging from 1 to 100%, to quantify the effect of heterogeneity [30]. *P ≤* 0.10 was deemed to represent significant heterogeneity [31, 32], and pooled RR was estimated using a random-effect model (the DerSimonian and Laird method [33]). On the contrary, if statistical study heterogeneity was not observed (*P* ≥ 0.10), a fixed effects model (the Mantel–Haenszel method [34]) was used. The effects of the risk factor of limb fracture in type 2 diabetes mellitus considered to be statistically significant if RRs 95% CI did not overlap with 1.

In addition, we performed subgroup analysis according to site of fracture like proximal humerus, distal forearm, leg or ankle, wrist/hand/foot and other limb fractures. Besides, sensitivity analysis was performed to examine the stability of the combined results. Finally, publication bias was assessed by contour-enhanced funnel plots. If the shape of funnel plots revealed no obvious evidence of asymmetry, we considered that there was no obvious publication bias. All statistical analyses were performed using standard statistical procedures provided in Review Manager 5.2 [29].

## SUPPLEMENTARY MATERIALS FIGURES AND TABLES




