# Supplementary material for: The association between risk of limb fracture and type 2 diabetes mellitus
Source: Oncotarget. 2018 Jan 5;9(58):31302–10. doi: 10.18632/oncotarget.23937 (PMC6101281; doi:10.18632/oncotarget.23937)
Supplement: Supplementary file 1 [file oncotarget-09-31302-s001.pdf]

The association between risk of limb fracture and type 2 diabetes mellitus

SUPPLEMENTARY MATERIALS

Supplementary Table 1: The characteristics of included studies for meta-analysis. See Supplementary_Table_1
