# Supplementary material for: The association between risk of limb fracture and type 2 diabetes mellitus
Source: Oncotarget. 2018 Jan 5;9(58):31302–10. doi: 10.18632/oncotarget.23937 (PMC6101281; doi:10.18632/oncotarget.23937)
Supplement: Supplementary file 2 [file oncotarget-09-31302-s002.docx]

**Supplementary Table 1.** The characteristics of included studies for meta-analysis.

| Author/Year | Country | Study design | | Age (year)* | Total number of Participants | | Number of Women | Number of Men | | | Number of  diabetes | | Follow-up time (year)* | Site of fracture | Adjustment for covariates | | NOS score | |
| --- | --- | --- | --- | --- | --- | --- | --- | --- | --- | --- | --- | --- | --- | --- | --- | --- | --- | --- |
| Bonds et al. 2006 | USA | Cohort | | 64.9±7 | 93,405 | | 93,405 | ‒ | | | 5,285 | | 7 | Hip, pelvis, leg, ankle, knee, foot, arm, shoulder, elbow, wrist, hand, spine | | Age; ethnicity; weight; height; time-dependent history of falls; previous fracture; history of osteoporosis; trouble seeing at baseline; alcohol or tobacco use; calcium and vitamin D intake; exercise; bisphosphonate, estrogen, steroid, insulin, thyroid hormone use. | | 7 |
| de Liefde et al. 2005 | Netherlands | Cohort | | ≥55 | 6,655 | | 3,964 | 2,691 | | | 792 | | 6.3±2.3 | Nonvertebral, hip, wrist | | Age, gender, BMI, smoking, serum creatinine, visual acuity, falling frequency and lower limb disability. | | 7 |
| Gerdhem et al. 2005 | Sweden | Cohort | | 75 | 1,028 | | 1,028 | ‒ | | | 67 | | 4.6 | Any type or hip, forearm or vertebral | | Body weight, bone mass and bone markers. | | 7 |
| Heath et al. 1980 | USA | | Cohort | <103 | | 20,897 | NR | | NR | 986 | | NR | | Osteoporotic, non-osteop-  orotic | | Use of insulin, sex and date of diagnosis of Diabetes Mellitus. | | 6 |
| Holmberg et al. 2006 | Sweden | Cohort | | 27–68 | 33,346 | | 1,878 | 2,422 | | | 166 | | 17 | Forearm, vertebral, proximal humerus, ankle, hip | Age, BMI, DBP, resting pulse rate, triglyceride level, gamma-glutamyltransferase, smoking, poor self-rated health, sedimentation rate for women, and cholesterol or creatinine for men. | | 8 | |
| Ivers et al. 2001 | Australia | Cohort | | 49–97 | 3654 | | NR | NR | | | 216 | | 5 | Hip, distal forearm, ankle, proximal humerus | | Age, sex and BMI. | | 6 |
| Keegan et al. 2002 | USA | Case-control | | ≥45 | 4,528 | | 3,412 | 1,116 | | | 531 | | NR | Distal forearm, foot, proximal humerus, tibia/fibula, pelvis | | Age, gender, Self-reported race/ethnicity (Black, Hispanic, Asian), Type of interview (telephone or in person). | | 7 |
| Melton et al. 2008 | USA | Cohort | | 30–97 | 1964 | | 973 | 992 | | | 700 | | 11.8 | Skull/face, hands/fingers, distal forearm, proximal humerus, proximal femur | | Age, sex, prior osteoporotic fracture, physically active, BMI, secondary osteoporosis, falling factors, neuropathy, use of insulin, biquanides, corticosteroids, osteoporosis drugs and anticoagulants. | | 8 |
| Oei et al. 2013 | Netherlands | Cohort | | ≥55 | 4135 | | NR | NR | | | 420 | | 12.2 | Hip, wrist | | Age, sex, height, and weight, and femoral neck BMD. | | 6 |
| Schwartz et al. 2001 | USA | Cohort | | ≥65 | 9654 | | 9654 | 0 | | | 657 | | 9.4 | Hip, proximal humerus, distal forearm, ankle, foot, vertebral | Age, BMI, BMD, height, height loss since age 25, contrast sensitivity, walking speed, alcohol, resting pulse. use of benzodiazepines, calcium intake | | 8 | |
| Vestrgaad et al. 2005 | Denmark | Case-control | | 43±27 | 484,657 | | 261,052 | 223,605 | | | 124,655 | | NR | Hip, forearm, spine | Prior fracture, use of corticosteroid, anti-epileptic drugs, diuretics, anxiolytics, sedatives, neuroleptics, antidepressants, ntihypertensives, statins, or non-statin cholesterol-lowering drugs, oralcoholism, MI, stroke, income, living with another person. | | 7 | |

Notes: *Data shown as mean, range, or mean ± SD; BMI, body mass index; BMD, bone mineral density; DBP, diastolic blood pressure; MI, myocardial infarction; NOS, Newcastle-Ottawa Scale; NR, not report.
